# Isolation of dihydrobenzofuran derivatives from ethnomedicinal species *Polygonum barbatum* as anticancer compounds

**DOI:** 10.1186/s40659-018-0209-0

**Published:** 2019-01-07

**Authors:** Umar Farooq, Sadia Naz, Afshan Shams, Yasir Raza, Ayaz Ahmed, Umer Rashid, Abdul Sadiq

**Affiliations:** 1 Department of Chemistry, COMSATS University Islamabad, Abbottabad Campus, Abbottabad, 22060 Pakistan; 2 Beijing National Laboratory for Molecular Sciences, State Key Laboratory of Molecular Reaction Dynamics, Institute of Chemistry, Chinese Academy of Sciences, Beijing, 100190 China; 3 Dr. Panjwani Center for Molecular Medicine and Drug Research, International Center for Chemical and Biological Sciences, University of Karachi, Karachi, Pakistan; 4 Department of Microbiology, University of Karachi, Karachi, Pakistan; 5 Department of Pharmacy, University of Malakand, Dir (L), Chakdara, 18000 KP Pakistan; 6 Department of Life Sciences & Chemistry, Faculty of Health, Jacobs University Bremen, 28759 Bremen, Germany

**Keywords:** Dihydrobenzofuran, *Polygonum barbatum*, Ethnomedicine, Oral and lungs carcinoma, Antiangiogenesis, Anticancer

## Abstract

**Background:**

Ethnomedicinally, the family Polygonaceae is famous for the management of cancer. Various species of this family have been reported with anticancer potentials. This study was designed to isolate anticancer compounds from ethnomedicinally important species *Polygonum barbatum*.

**Methods:**

The column chromatography was used for the isolation of compounds from the solvent fraction of *P. barbatum*. The characterization of isolated compounds was performed by various spectroscopic techniques like UV, IR, mass spectrometry and 1D-2D NMR spectroscopy. Keeping in view the ethnomedicinal importance of the family, genus and species of *P. barbatum*, the isolated compounds (**1**–**3**) were screened for anticancer potentials against oral cancer (CAL-27) and lungs cancer (NCI H460) cell lines using MTT assay. Active compound was further investigated for apoptosis by using morphological changes and flow cytometry analysis. In vivo anti-angiogenic study of the isolated compounds was also carried using chorioallantoic membrane assay. Docking studies were carried out to explore the mechanism of anticancer activity.

**Results:**

Three dihydrobenzofuran derivatives (**1**–**3**) have been isolated from the ethyl acetate fraction of *P. barbatum*. The structures of isolated compounds were elucidated as methyl (2*S*,3*S*)-2-(3,4-dimethoxyphenyl)-4-((*E*)-3-ethoxy-3-oxoprop-1-en-1-yl)-7-methoxy-2,3-dihydrobenzo-furan-3-carboxylate (**1**), (*E*)-3-((2*S*,3*S*)-2-(3,4-dimethoxyphenyl)-7-methoxy-3-(methoxy carbonyl)-2,3-dihydrobenzofuran-4-yl)acrylic acid (**2**) and (2*S*,3*S*)-4-((*E*)-2-carboxyvinyl)-2-(3,4-dimethoxyphenyl)-7-hydroxy-2,3-dihydrobenzofuran-3-carboxylic acid (**3**). The compound **1** was found to be more potent with IC_50_ of 48.52 ± 0.95 and 53.24 ± 1.49 against oral cancer cells as compared to standard drug (IC_50_ = 97.76 ± 3.44 μM). Both compound also inhibited lung cancer cells but at higher concentrations. Morphological and flow cytometry analysis further confirms that compound **1** induces apoptosis after 24 to 48 h treatment. In antiangiogenesis assay, compounds **1**, **2** and **3** exhibited IC_50_ values of 8.2 ± 1.1, 13.4 ± 1.1 and 57.7 ± 0.3 μM respectively. The docking studies revealed that the compounds under study have the potential to target the DNA and thymidylate synthase (TS).

**Conclusion:**

Based on its overwhelming potency against the tested cell lines and in angiogenesis assay, compound **1** can be further evaluated mechanistically and can be developed as anticancer drug candidate.

**Electronic supplementary material:**

The online version of this article (10.1186/s40659-018-0209-0) contains supplementary material, which is available to authorized users.

## Introduction

*Polygonum barbatum* is a perennial herb, mostly found in shady and marshy places near river banks, ponds and other aquatic areas in different countries of South-East Asia [25, 26]. The species belongs to the family Polygonaceae represented by 19 genera and 103 species in Pakistan out of total 55 genera and approximately 1200 species worldwide [21]. Traditionally, various species of Polygonaceae family are used for the management of cardiovascular diseases, hypertension and cancer [14, 29]. Based on the ethnomedicinal background, some species have been verified scientifically for the management of cancer [1, 2, 4, 17]. The leaf extract of *P. barbatum* has been used in Chinese traditional medicine for the treatment of ulcer, and root extract as astringent and as carminative agent [10, 20, 30].

Anti-inflammatory, antinociceptive and diuretic activities of *P. barbatum* has been previously reported [10, 25]. Similarly, *P. barbatum* also possess potential analgesic, brine shrimps, cholinergic, as well as spasmolytic potentials [10, 11, 24]. In indigenous system of medicine, it has been used as potential anticancer agent against oral cancer [18]. *P. barbatum* has also been reported to have antimicrobial activity, suggesting it to be a source of potential antibiotic agents [18].

Additionally, the therapeutic and biological potentials of some species of the genus Polygonum have also been previously reported [5–8, 19, 21, 22]. Phytochemical investigations of various species of this genus showed the presence of secondary metabolites, particularly anthraquinones, flavonoids, as well as phenylpropanoid [9, 16]. Furthermore, secondary metabolites such as acetophenone, viscozulenic acid and sitosterone have been previously isolated from *P. barbatum* [26]. Similarly, Farooq et al. reported sesquiterpenes from *P. barbatum* possessing potential anti-proliferative activity and inhibitory effect on cancer cell migration [13].

Cancer, a second leading cause of death, resulted in 8.8 million deaths in 2015 globally [32]. In Pakistan, the three deadliestof them are breast cancer, oral cancer and lungs cancer with death rate of 26.76, 8.71 and 5.53 per 100,000 population, respectively [33]. Despite of the current advances in therapeutic approaches, there is an immense need of new potential compounds that can be used as safe anticancer drugs. Keeping in view the ethnomedicinal importance of Polygonaceae and potential therapeutic efficiency of *P. barbatum*, we were able to isolate three dihydrobenzofuran derivatives from its ethyl acetate fraction. To study the anticancer effect, we targeted oral squamous cell carcinoma and large cell lung carcinoma. Furthermore, we also assessed the potential of these isolated dihydrobenzofuran derivatives on normal vascular development in chick embryos using a multifaceted and low-cost chicken chorioallantoic membrane (CAM) assay.

## Methods

The *Polygonum barbatum* (whole plant) was collected from different areas of Khyber Pakhtunkhwa in October 2015 and specimen having voucher # 66130 has been deposited in herbarium at Department of Botany, postgraduate college Abbottabad, Pakistan.

The shade dried plant (5.4 kg) was ground into fine powder, extracted with methanol and filtered twice. The crude extract (245 g) was obtained from filtrate by using vacuum rotary evaporator and partitioned into *n*-hexane (85 g), ethyl acetate (48 g) and *n*-butanol (94 g) fraction. Further investigation and purification of compounds was done through column chromatography of ethyl acetate fraction selected based on TLC.

### Column chromatography

The column chromatography of ethyl acetate fraction was carried out using *n*-hexane/ethyl acetate as solvent with increasing polarity of ethyl acetate up to 100%, later followed by methanol. Various sub-fractions (1–12) were obtained as a result of column chromatography and sub-fractions (4–12) were re-selected on the basis of TLC and subjected to re-column chromatography to purify compounds (**1**–**3**). The compound **1** (7 mg) was obtained at polarity *n*-hexane:ethyl acetate (65:35) from sub-fractions 4–10 while compound **2** and **3** were obtained from re-column chromatography of sub-fractions (11–12) at polarity of *n*-hexane:ethyl acetate (58:42) and (55:45) respectively.

### Instrumentation

The NMR spectra were recorded by using Bruker AMX-400 spectrometer (^1^H-NMR at 400 MHz and ^13^C-NMR at 100 MHz). The values of chemical shift and coupling constant were recorded as ppm and Hertz (Hz) respectively. The Varian MAT-312 spectrometer was used to record HR-EI-MS and EI-MS spectra. The E. Merck 230–400 mesh silica gel was used to pack columns while pre-coated silica gel plates were employed for TLC analysis. In addition, Ceric sulphate solution in 10% sulphuric acid (H_2_SO_4_) has been used for detection of UV active compounds.

#### Methyl (2S,3S)-2-(3,4-dimethoxyphenyl)-4-((E)-3-ethoxy-3-oxoprop-1-en-1-yl)-7-methoxy-2,3-dihydrobenzofuran-3-carboxylate (**1**)

A yellow gum; UV λ_max_: 306 (2.4), 220 (3.4) nm; IR (KBr) ʋ_max_: 3036, 2810, 1734, 1440, 1204 cm^−1^; HR-EI-MS (m/z): Calcd. 442.1628, mol. formula C_24_H_26_O_8_; Obs. 442.1621; EI-MS (m/z): 442, 414, 396, 370, 338, 306, 264, 180, 140, 126, 96; [∝]_*D*_^25^ + 60 (c 0.39, CHCl_3_); ^1^H NMR (400 MHz, CD_3_OD) δ_H_ (ppm): 6.90 (d, *J* = 9.1 Hz, H-5, 1H), 7.30 (d, *J* = 9.1 Hz, H-6, 1H), 7.80 (d, *J* = 16.5 Hz, H-7, 1H), 6.54 (d, *J* = 16.5 Hz, H-8, 1H), 4.30 (q, *J* = 8.1 Hz, H-10, 2H), 1.10 (t, *J* = 8.1 Hz, H-11, 3H), 6.80 (d, *J* = 1.8 Hz, H-2′, 1H), 6.61 (d, *J* = 8.2 Hz, H-5′, 1H), 6.63 (dd, *J* = 8.2, 1.8 Hz, H-6′, 1H), 5.92 (d, *J* = 5.2 Hz, H-7′, 1H), 4.68 (d, *J* = 5.2 Hz, H-8′, 1H), 3.60 (s, 4-OCH_3_, 3H), 3.64 (s, 3′-OCH_3_, 3H), 3.66 (s, 4′-OCH_3_, 3H), 3.55 (s, 9′-OCH_3_, 3H); ^13^C NMR (100 MHz, CD_3_OD) δ_C_ (ppm): 126.6 (C-1), 129.7 (C-2), 151.9 (C-3), 146.3 (C-4), 121.3 (C-5), 122.4 (C-6), 143.7 (C-7), 120.7 (C-8), 170.1 (C-9), 66.2 (C-10), 19.4 (C-11), 135.9 (C-1′), 116.3 (C-2′), 153.2 (C-3′), 154.5 (C-4′), 118.6 (C-5′), 124.8 (C-6′), 82.4 (C-7′), 58.1 (C-8′), 174.6 (C-9′), 57.8 (4-OCH_3_), 60.1 (3′-OCH_3_), 60.5 (4′-OCH_3_), 54.8 (9′-OCH_3_).

#### (E)-3-((2S,3S)-2-(3,4-dimethoxyphenyl)-7-methoxy-3-(methoxycarbonyl)-2,3-dihydrobenzofuran-4-yl) acrylic acid (**2**)

A yellowish-brown gummy solid; UV λ_max_: 308 (1.8), 218 (2.6), 202 (3.2) nm; IR (KBr) ʋ_max_: 3020, 2822, 1743, 1710, 1610, 1540, 1201 cm^−1^; HR-EI-MS (m/z): Calcd. 414.1315, mol. formula C_22_H_22_O_8_; Obs. 414.1304; EI-MS (m/z): 414, 396, 368, 336, 304, 182, 140, 126, 96; [∝]_*D*_^25^ +46 (c 0.190, CHCl_3_);^1^H NMR (400 MHz, CD_3_OD) δ_H_ (ppm): 6.88 (d, *J* = 9.2 Hz, H-5, 1H), 7.28 (d, *J *= 9.2 Hz, H-6, 1H), 7.70 (d, *J* = 15.4 Hz, H-7, 1H), 6.50 (d, *J* = 15.4 Hz, H-8, 1H), 6.80 (d, *J* = 1.9 Hz, H-2′, 1H), 6.65 (d, *J* = 8.8 Hz, H-5′, 1H), 6.72 (dd, *J* = 8.8, 1.9 Hz, H-6′, 1H), 5.90 (d, *J* = 4.8 Hz, H-7′, 1H), 4.70 (d, *J* = 4.8 Hz, H-8′, 1H), 3.60 (s, 4-OCH_3_, 3H), 3.62 (s, 3′-OCH_3_, 3H), 3.65 (s, 4′-OCH_3_, 3H), 3.53 (s, 9′-OCH_3_, 3H); ^13^C NMR (100 MHz, CD_3_OD) δ_C_ (ppm): 124.5 (C-1), 128.2 (C-2), 150.6 (C-3), 147.8 (C-4), 120.2 (C-5), 125.1 (C-6), 142.4 (C-7), 119.6 (C-8), 172.1 (C-9), 134.7 (C-1′), 114.3 (C-2′), 152.7 (C-3′), 153.1 (C-4′), 117.6 (C-5′), 123.1 (C-6′), 84.1 (C-7′), 59.2 (C-8′), 174.1 (C-9′), 58.6 (4-OCH_3_), 61.3 (3′-OCH_3_), 61.6 (4′-OCH_3_), 56.4 (9′-OCH_3_).

#### (2S,3S)-4-((E)-2-carboxyvinyl)-2-(3,4-dimethoxyphenyl)-7-hydroxy-2,3-dihydrobenzofuran-3-carboxylic acid (**3**)

A brown gummy solid; UV λ_max_: 320 (2.1), 270 (3.1), 210 (1.9) nm; IR (KBr) ʋ_max_: 3370, 2950, 2820, 1710, 1640, 1480, 1240 cm^−1^; HR-EI-MS (m/z): Calcd. 386.1002, mol. formula C_20_H_18_O_8_; Obs. 386.997; EI-MS (m/z): 386, 368, 350, 342, 338, 306, 184, 140, 126, 96; [∝]_*D*_^25^ +80 (c 0.172, CHCl_3_);^1^H NMR (400 MHz, CD_3_OD) δ_H_ (ppm): 6.89 (d, *J* = 8.8 Hz, H-5, 1H), 7.27 (d, *J* = 8.8 Hz, H-6, 1H), 7.78 (d, *J* = 17.1 Hz, H-7, 1H), 6.48 (d, *J* = 17.1 Hz, H-8, 1H), 6.74 (d, *J* = 2.1 Hz, H-2′, 1H), 6.64 (d, *J* = 7.9 Hz, H-5′, 1H), 6.68 (dd, *J* = 7.9, 2.1 Hz, H-6′, 1H), 5.91 (d, *J* = 5.5 Hz, H-7′, 1H), 4.74 (d, *J* = 5.5 Hz, H-8′, 1H), 3.58 (s, 3′-OCH_3_, 3H), 3.60 (s, 4′-OCH_3_, 3H); ^13^C NMR (100 MHz, CD_3_OD) δ_C_ (ppm): 124.2 (C-1), 128.1 (C-2), 140.4 (C-3), 146.6 (C-4), 119.9 (C-5), 123.1 (C-6), 141.4 (C-7), 120.8 (C-8), 173.1 (C-9), 135.7 (C-1′), 118.6 (C-2′), 153.1 (C-3′), 154.6 (C-4′), 116.7 (C-5′), 125.3 (C-6′), 84.4 (C-7′), 57.1 (C-8′), 175.6 (C-9′), 59.1 (3′-OCH_3_), 59.8 (4′-OCH_3_).

### Anticancer activity

The isolated compounds (**1**–**3**) were investigated for anticancer activity against human oral squamous cell carcinoma cell line (CAL 27) and human large cell lung carcinoma cell line (NCI H460) as described recently [13]. The cell lines were purchased from ATCC and maintained in Dulbecco’s Modified Eagle’s Medium (DMEM) supplemented with 10% Fetal Bovine Serum (FBS), 100 µg/mL streptomycin and 100 units/mL penicillin at 37 °C in a humidified 5% CO_2_ incubator. The CAL 27 and NCI H460 cells were seeded in 96 well plates with the density of 1.5 × 10^4^ and 1 × 10^4^ cells per well, respectively and incubated for 24 h. The cells were then treated with different concentrations of compound **1**, **2** and **3** for 48 h. 1% DMSO was used as vehicle control and empty wells were used as negative control. The standard anticancer drugs namely 5-Fluorouracil and Cisplatin were used against CAL 27 and NCI H460, respectively. Quantification of metabolically active cells were determined using 3-(4,5-dimethyl-2-thiazolyl)-2,5-diphenyl-2-H-tetrazolium bromide (MTT) dye which reduces to insoluble Formazan crystals by the action of mitochondrial succinate dehydrogenase. The soluble MTT dye was aspirated off and insoluble crystals were dissolved using DMSO. The optical density of each well was determined by SpectraMax microplate reader at 570 nm. IC_50_ values were calculated using software EZ-Fit Enzyme Kinetics by Perrella Scientific. For morphological changes cells treated at IC_50_ and IC_70_ were observed under phase contrast microscopy (Nikon, Japan) at 24 and 48 h respectively.

### YO-PRO-1 apoptotic assay

Oral cancer (CAL-27) cells (5 × 10^5^) were seeded in a 6-well plate and incubated for 24 h in presence of 5% CO_2_. After incubation compound **1** was added at IC_50_ and IC_70_ concentration and further incubated for 48 h. Following incubation cells were harvested by using trypsin (0.05%) and pallet out after washing three times at 400×*g* for 5 min to remove media and trypsin. Cell pallets were suspended in 1 mL PBS and stained with Vybrant Apoptosis Assay kit # 4 (Invitrogen, USA) containing YO-PRO-1 and Propidium Iodide (PI) dye according to manufacturer protocol. Briefly, cells were incubated with 1 μL YO-PRO-1 stock solution (Component A) and 1 μL PI stock solution (Component B) for 30 min on ice. Later, cells were analyzed on FACSCalibur (Becton–Dickinson, USA). YO-PRO-1 and PI were excited at 488 nm, and fluorescence was measured at 530 and 620 nm, respectively. A total of 10,000 events were acquired from each sample. The percentages of live, apoptotic and dead cells were determined using CellquestPro software.

### Chorioallantoic membrane (CAM) assay

Chorioallantoic membrane (CAM) assay was performed according to our previously reported procedure [15]. The experiments were carried out as per the approval of the ethical committee, Department of Pharmacy, University of Malakand, Pakistan according to the animals Bye-Laws 2008 (Scientific Procedure Issue-1). The fertilized domestic chicken eggs were purchased from poultry form Chakdara, Pakistan. The fertilized eggs were incubated for 5–6 days at 37 °C with slowly shaking for at least two to three times a day. After the incubation period, the seven-day old eggs were examined under the flash light for detection of the embryo head. After that a small hole was bored at the narrow end of the eggs and 0.5–1 mL of albumin was sucked with the help of eighteen-gauge hypodermic needle so that yolk sacs fell down from the shell membrane. The shell around the embryo air sac was removed through forceps and the shell membrane at the base of air sac was peel away. On 8th day, a thermanox cover slip was carefully placed on the surface of CAM loaded with different samples for each concentration (10 µL) and were placed in incubator. After 24 to 48 h, the numbers of vessels on the surface of CAM were observed. The numbers of blood vessels in each egg was counted by figuring out the actual number of blood vessels in 1 cm^2^ area of the CAM adjacent to the sample application site. Vessels radially converging in the direction of the center were counted under a microscope. At least twenty eggs were used for each sample dose. The % of increase and inhibition were calculated using formula;

$${\text{\% inhibition}} = \frac{{{\text{No}}.\;{\text{of blood vessels }}\left( {\text{Norml Saline}} \right) - {\text{No}}.\;{\text{of blood vessles }}\left( {\text{Test Sample}} \right)}}{{{\text{No}}.{\text{of blood vessels }}\left( {\text{Norml Saline}} \right)}} \times 100$$

Median inhibitory concentrations (IC_50_) for anti-angiogenic assay were calculated via regression analysis of dose response curve by adding all the concentration and percent activity.

### Docking studies

Docking studies were carried out using Molecular Operating Environment [27] software. Crystal structures of DNA and Human Thymidylate Synthase (TS) was retrieved from Protein Data Bank with code numbers 3LPV and 1JU6 respectively. Validation of docking procedure was carried out by using re-dock method. Ligand and DNA/enzyme preparation, active site determination and docking procedure was carried out by using our previously reported procedures [3, 15]. The lowest energy minimized pose was used for further analysis. Ligand-interaction module of MOE was used to calculate the 2D ligand–enzyme interactions. The view of the docking results and analysis of their surface with graphical representations were done using MOE and discovery studio visualizer [12].

## Results and discussion

### Isolation and Characterization of benzofuran derivatives

The ethyl acetate fraction of *P. barbatum* upon column chromatography gave three benzofuran derivatives (1–3). Using various spectroscopic analyses and published literature, the structures of isolated compounds were determined.

#### Compound **1**

Compound **1**, a white gummy solid having molecular formula C_24_H_26_O_8_ as suggested by molecular ion peak of 442.1621 in HR-EI-MS was obtained by repeated chromatography. Other m/z peaks were obtained at 442, 414, 396, 370, 338, 306, 264, 180, 140, 126, and 96. The UV spectrum showed absorption bands at λ_max_ 306 (2.4) and 220 (3.4) nm while IR spectrum showed absorption at 3036, 2810, 1734, 1440, 1204 cm^−1^ suggesting the presence of alkene, ester, and alkyl aryl ether moiety.

The ^1^H-NMR spectrum showed two ortho coupled proton with chemical shift value of δ_H_ 6.90 (d, *J* = 9.1 Hz, H-5, 1H) and 7.30 (d, *J* = 9.1 Hz, H-6, 1H). Similarly, two doublets for other aromatic protons were also revealed in ^1^H-NMR spectrum at δ_H_ 6.80 (d, *J* = 1.8 Hz, H-2′, 1H), δ_H_ 6.61 (d, *J* = 8.2 Hz, H-5′, 1H,) while doublet of doublet was observed for proton at δ_H_ 6.63 (dd, *J* = 8.2, 1.8 Hz, H-6′, 1H). The coupling observed between two aliphatic protons at δ_H_ 5.92 (d, *J* = 5.2 Hz, H-7′, 1H) and δ_H_ 4.68 (d, *J* = 5.2 Hz, H-8′, 1H) suggested the presence of dihydrobenzofuran ring [34]. The protons at position H-7′ and H-8′ were suggested at trans position to each other as indicated by value of coupling constant, i.e. 5.2 Hz. The ^1^H-NMR also revealed the presence of two olefinic protons at δ_H_ 7.80 (d, *J* = 16.5 Hz, H-7, 1H) and δ_H_ 6.54 (d, *J* = 16.5 Hz, H-8, 1H) evident from trans-olefinic protons with extended conjugation with carbonyl group. A quartet was observed for methylene protons at position H-10 (δ_H_ 4.30, q, *J* = 8.1 Hz) while the terminal methyl group at position H-11 (δ_H_ 1.10, t, *J* = 8.1 Hz) was directly connected with methylene group. In addition, four singlets for methoxy group (–OCH_3_) were observed in ^1^H-NMR spectrum at chemical shift values of δ_H_ 3.60 (3H, s, H-4), δ_H_ 3.64 (3H, s, H-3′), δ_H_ 3.66 (3H, s, H-4′), and δ_H_ 3.55 (3H, s, H-9′) (Table S1 in Additional file 1).

**Fig. 1:**
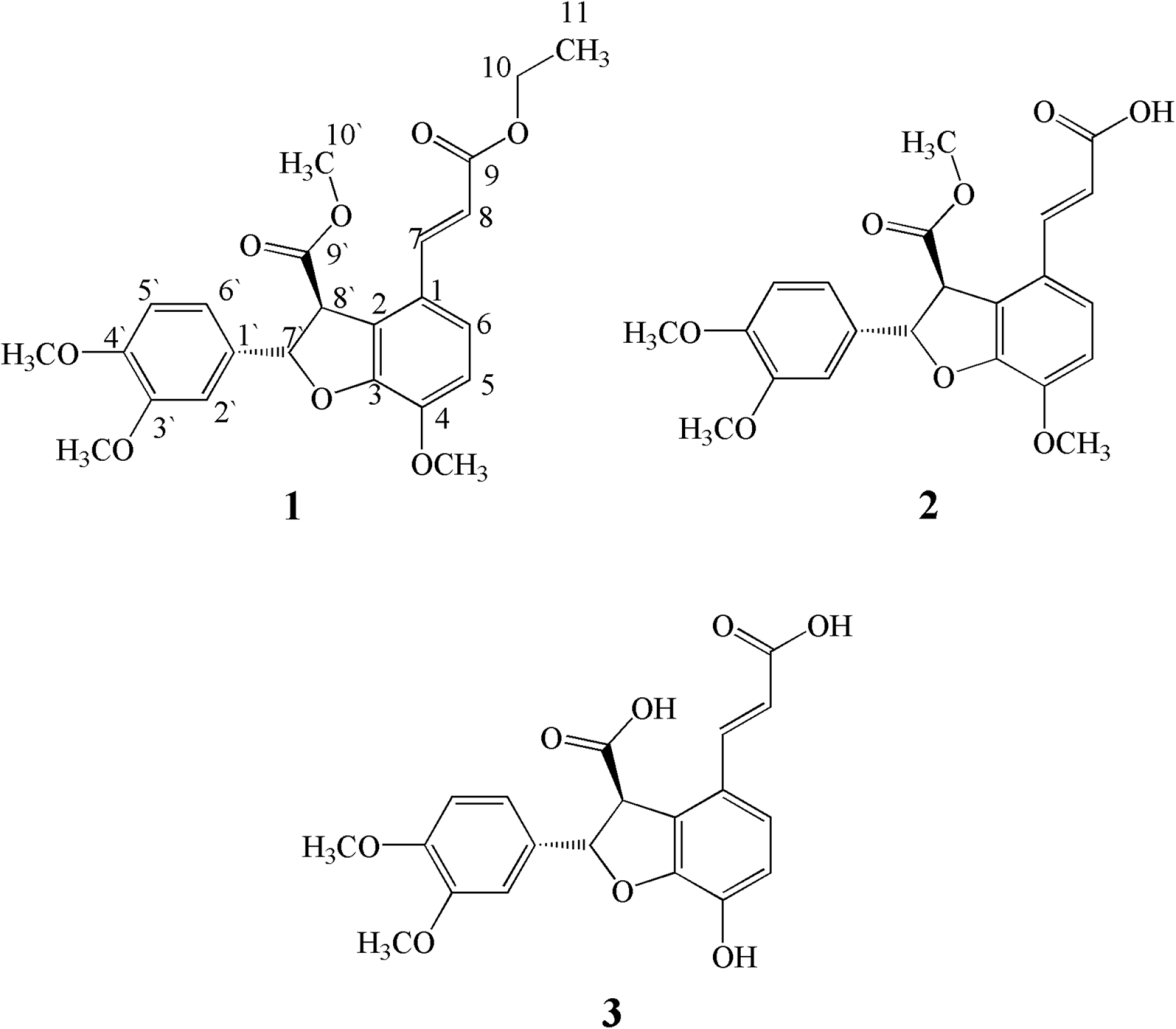
Structure of compounds **1**–**3**

The ^13^C-NMR along with DEPT spectrum showed signals for 24 carbon atoms comprising of one methyl, one methylene, nine methine, seven quaternary and four methoxy groups. In addition, two carbonyl carbon atoms were observed at δ_C_ 170.1 (C-9) and δ_C_ 174.6 (C-9′) while the olefinic carbons at position H-7 and H-8 resonated at δ_C_ 143.7 and 120.7 respectively (as shown in Fig. 1). The only downfield methylene appeared at δ_C_ 66.2, while the methyl group resonated at δ_C_ 19.4 ppm. The two methine carbons of dihydrobenzofuran centered at δ_C_ 82.4 for C-7′ and δ_C_ 58.1 for C-8′. The chemical shift values of aromatic carbon and methoxy groups have been shown in Additional file 1: Table S2.

**Fig. 2:**
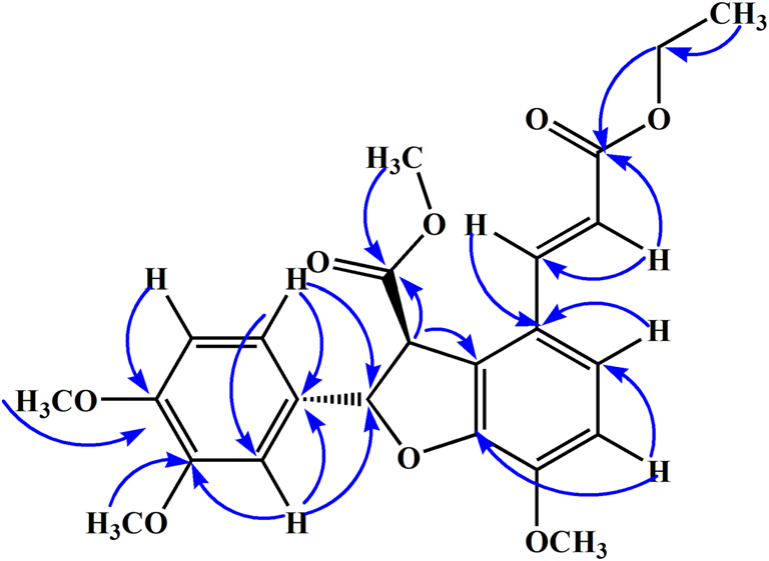
Important HMBC correlation of compound **1**

The HMBC spectrum revealed strong interaction of protons at H-5 and H-6 with C-4 and C-1 while protons of methoxy group substituent at this position (C-4) also showed strong correlation with C-4. The olefinic proton at position H-7 showed strong HMBC correlation with C-1 and C-8 while other olefinic proton at H-8 showed HMBC correlation with C-1, C-7 and C-9 (carbonyl carbon of ethyl ester moiety). The placement of methyl ester as substituent on dihydrobenzofuran ring was confirmed by HMBC correlation of methoxy protons were connected with carbonyl carbon at C-9′ as evident from Fig. 2. This placement was further supported by H-7′ and H-8′ protons which showed strong correlations with C-9′ carbon. The position of two methoxy group at position 3′ and 4′ was also confirmed by their HMBC correlation as depicted in Fig. 2. The aromatic protons at position 2′ and 6′ showed strong correlation with C-1′ and C-7′ confirming the placement of this aromatic ring at dihydrobenzofuran ring.

The relative configuration of aliphatic protons at position H-7′ and H-8′ was suggested trans to each other by Nuclear Overhauser Effect (NOE) as well as the coupling constant value (i.e., 5.2 Hz) suggested them to be at Trans position to each other. All the spectral data of ^1^H-NMR, ^13^C-NMR, HR-EI-MS, HMBC, and COSY along with comparison of previous literature the structure of compound **1** was confirmed as methyl (2*S*,3*S*)-2-(3,4-dimethoxyphenyl)-4-((*E*)-3-ethoxy-3-oxoprop-1-en-1-yl)-7-methoxy-2,3-dihydrobenzofuran-3-carboxylate.

#### Compound **2**

Compound **2** was isolated as yellowish gummy solid and was assigned molecular formula C_22_H_22_O_8_ as suggested by molecular ion peak of 414.1304 in HR-EI-MS while other peaks with m/z values were obtained at 414, 396, 368, 336, 304, 182, 140, 126 and 96. The UV absorption bands were observed at 308 (1.8), 218 (2.6) and 202 (3.2) nm suggesting the presence of alkene, carboxylic acid, ester and ether moieties which were further supported by IR absorption bands at 3020, 2822, 1743, 1710, 1610, 1540, 1201 cm^−1^.

The ^1^H-NMR spectrum showed doublet for proton at δ_H_ 6.65 (d, *J* = 8.8 Hz, H-5′, 1H) having ortho coupling with δ_H_ 6.72 (dd, *J* = 8.8, 1.9 Hz, H-6′, 1H) which was further coupled with δ_H_ 6.80 (d, *J* = 1.9 Hz, H-2′, 1H). Similarly, two ortho coupled aromatic protons of dihydrobenzofuran with chemical shift value of δ_H_ 6.88 (d, *J* = 9.2 Hz, H-5, 1H) and 7.28 (d, *J* = 9.2 Hz, H-6, 1H) were also observed in ^1^H-NMR quite similar to compound **1**. The two trans olefinic protons centered at δ_H_ 7.70 (d, *J* = 15.4 Hz, H-7, 1H) and 6.50 (d, *J* = 15.4 Hz, H-8, 1H) were also revealed by ^1^H-NMR spectrum. The two aliphatic protons at δ_H_ 5.90 (d, *J* = 4.8 Hz, H-7′, 1H) and 4.70 (d, *J* = 4.8 Hz, H-8′, 1H) were trans oriented to each other as suggested by their coupling constant value of 4.8 Hz [34]. The trans orientation of H-7′ and H-8′ was further supported by NOE experiment. When H-7′ was irradiated, H-8′ did not show any increase in intensity of H-8′ confirmed the trans orientation. In addition, singlets for methoxy groups were appeared at δ_H_ 3.60 (3H, s, 4-OMe), 3.62 (3H, s, 3′-OMe) and 3.65 (3H, s, 4′-OMe), while the methoxylgroup of ester moiety appeared at δ_H_ 3.53 as singlet in ^1^H-NMR spectrum.

The ^13^C-NMR spectra of compound **2** were quite identical to compound **1** that showed the presence of 22 carbon atoms including one methoxyl group of ester moiety, nine methine, seven quaternary and three methoxy groupson aromatic rings along with two carbonyl carbon atoms. The chemical shift value of olefinic carbon atoms were δ_C_ 142.4 (C-7) and 119.6 (C-8) while two carbonyl carbon resonated at δ_C_ 172.1 (C-9) and 174.1 (C-9′). The carbon atoms of five membered ring of dihydrobenzofuran resonated at δ_C_ 84.1 (C-7′) and 59.2 (C-8′) while chemical shift values of aromatic carbon atoms has been shown in Additional file 1: Table S2.

The placements of various substituents at different positions were assign with the help of HMBC spectrum and was quite identical to compound **1**. The methyl group at position 10′ showed HMBC correlation with C-9′ while aliphatic proton at C-8′ showed correlation with C-2, C-7′ and C-9′. The aromatic protons at position 2′ and 6′ showed strong correlation with 1′ and 7′ confirming the attachment of dihydrobenzofuran ring. In addition both olefinic protons (H-7 and H-8) showed strong HMBC correlation with C-9, and C-1. Finally, the structure of compound **2** was deduced as (*E*)-3-((2*S*,3*S*)-2-(3,4-dimethoxyphenyl)-7-methoxy-3-(methoxycarbonyl)-2,3-dihydrobenzofuran-4-yl)acrylic acid on the basis of all spectral data and literature comparison [34].

#### Compound **3**

Compound **3** was isolated as yellow gummy solid, that showed molecular ion peak of 386.997 in HR-EI-MS suggesting a molecular formula as C_20_H_18_O_8_. Other EI-MS peaks obtained were at m/z 386, 368, 342, 338, 306, 184, 140, 126 and 96. The absorption bands of UV spectrum were obtained at λ_max_ 320 (2.1), 270 (3.1) and 210 (1.9) nm. The IR spectrum showed absorption band at 3370, 2950, 2820, 1710, 1640, 1480 and 1240 cm^−1^ suggesting presence of different functional groups like hydroxyl group, alkene, carboxylic acid and ether moiety.

The ^1^H-NMR spectrum showed signals for 1, 2, 3, 4-tetra substituted benzene ring fused with furan ring at δ_H_ 6.89 (d, *J* = 8.8 Hz, H-5), 7.27 (d, *J *= 8.8 Hz, H-6) and two trans protons appeared at δ_H_ 5.91 (d, *J* = 5.5 Hz, H-7′), δ_H_ 4.74 (d, *J* = 5.5 Hz, H-8′) respectively. Similarly, trans-disubstituted double bond protons appeared at δ_H_ 7.78 (d, *J* = 17.1 Hz, H-7) and δ_H_ 6.48 (d, *J* = 17.1 Hz, H-8) with extended conjugation with carbonyl carbon of acid moiety.

Similarly,^13^C-NMR data was also in close agreement with those of compound (**1**–**2**). The ^13^C-NMR spectra suggested the presence of two carboxylic acid groups in compound **3**, which appeared at δ_C_ 173.1 (C-9) and δ_C_ 175.6 (C-9′). It was also evident that the fused benzene ring with furan ring was substituted with hydroxyl group at position 4 with chemical shift value of δ_C_ 146.6.

The ^1^H-NMR and ^13^C-NMR spectra of compound **3** were quite similar to compounds **1** and **2**. The differences observed were presence of carboxylic acid instead of ester substituent at position-8 while at position-4 a hydroxyl (–OH) group was observed as substituent rather than methoxy group. The difference was retrieved from UV, IR and NMR data which was further supported by mass spectrum. The chemical shift values of both ^1^H-NMR and ^13^C-NMR spectra have been given in Additional file 1: Tables S1 and S2. Similarly, HMBC correlation was also found similar to compound **1** and **2** as shown in Fig. 2. All spectra data and comparison of literature suggested the structure of compound **3** as (2*S*,3*S*)-4-((*E*)-2-carboxyvinyl)-2-(3,4-dimethoxyphenyl)-7-hydroxy-2,3-dihydrobenzofuran-3-carboxylic acid.

### In vitro anticancer activity

**Fig. 3:**
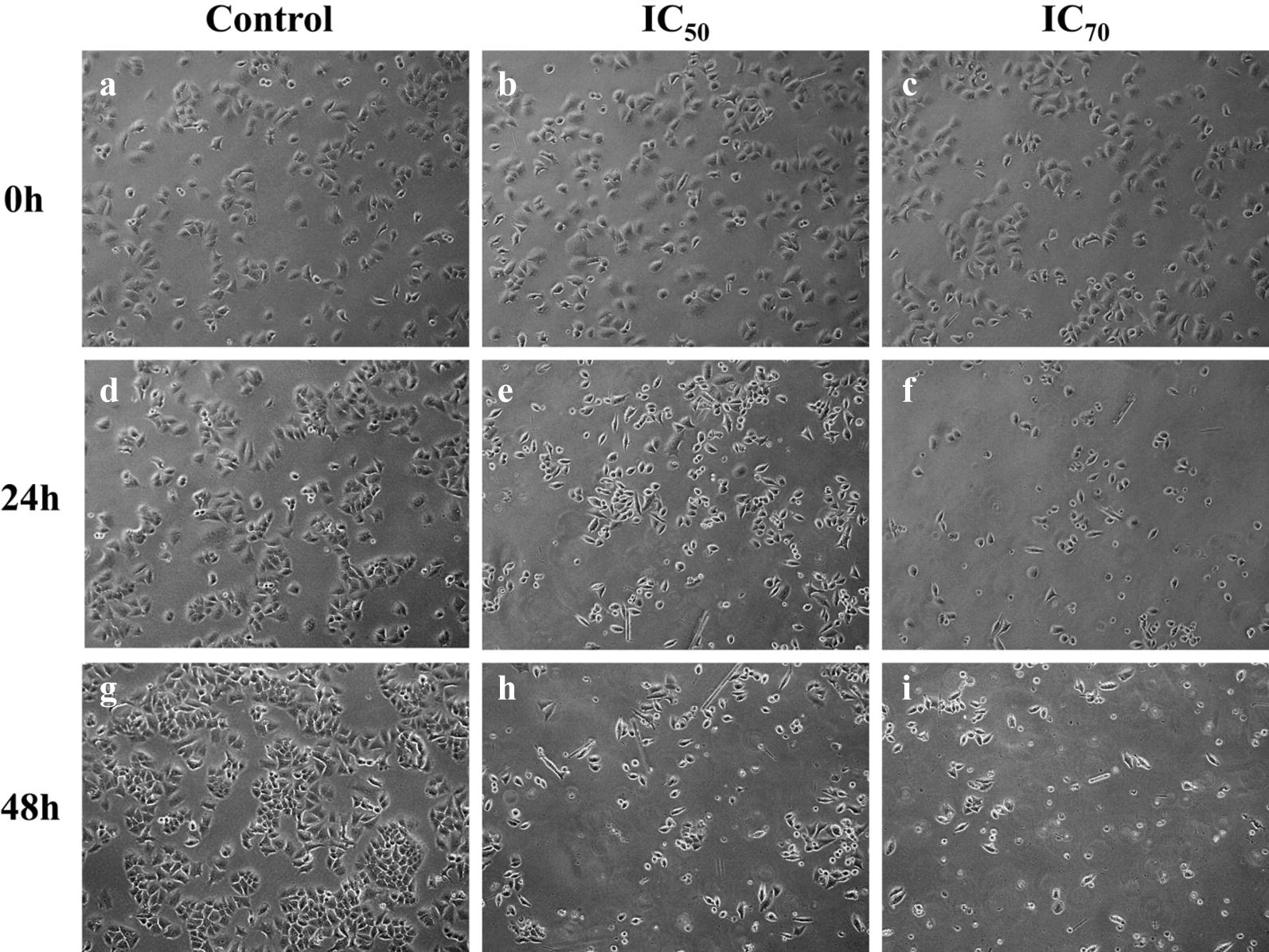
Morphological changes in CAL-27 cells after 24 and 48 h of treatment with compound **1** by using phase contrast microscope at ×10 magnification

**Fig. 4:**
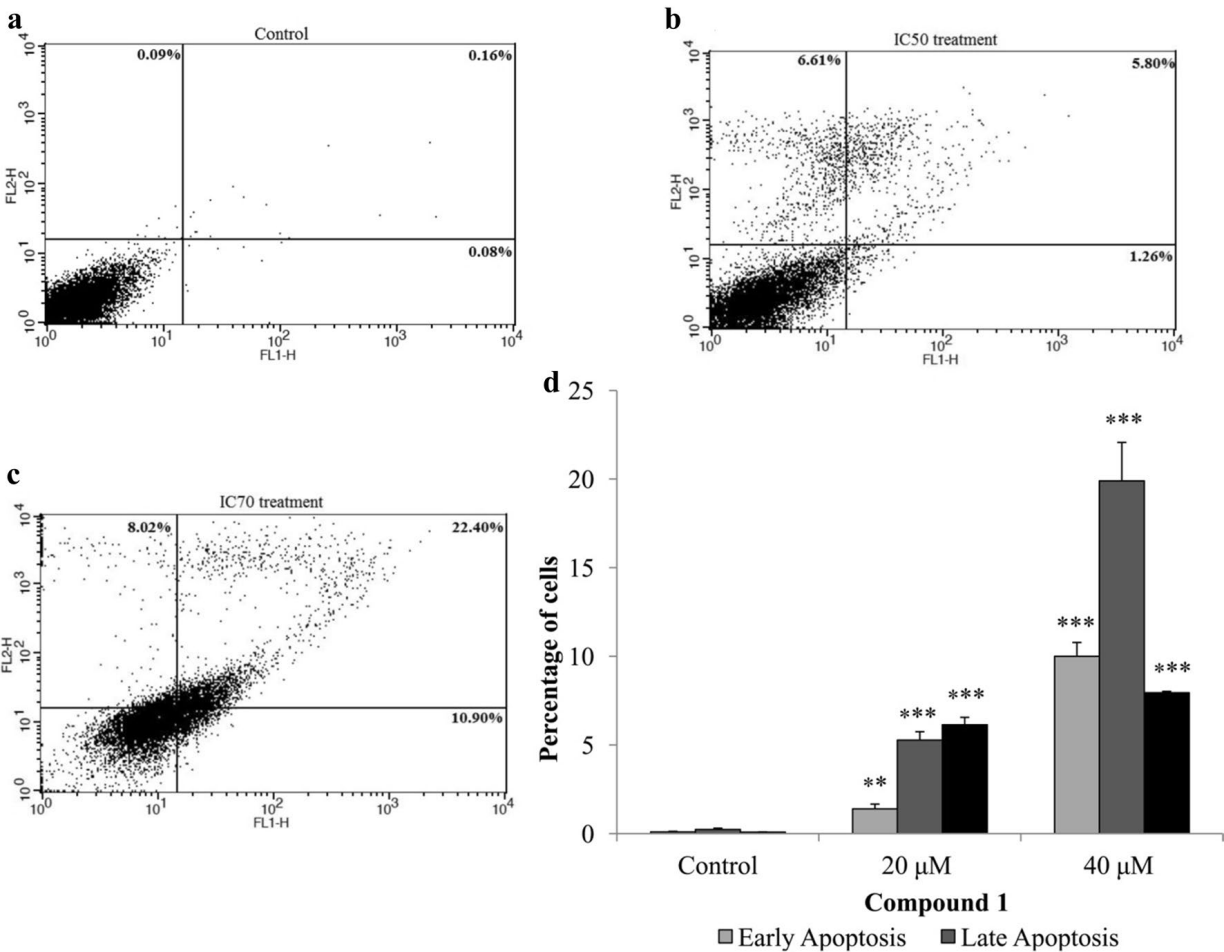
Compound **1** induced apoptosis in CAL-27 cell line in a dose-dependent manner. **a**–**c** Flow cytometric analysis of the apoptosis induced by the compound **1** at IC_50_ and IC_70_ concentrations. An increased number of apoptotic cells were observed after treatment as compared to control. **d** Graphical representation of the cells in different phases of apoptosis after 48 h of treatment. Statistical significance were expressed as **p <* 0.01* and ***p <* 0.001* as compared to the untreated control

As widely spread natural compounds, benzofuran derivatives have been extensively studied for their biological activities [23]. Previously, a series of synthetic dihydrobenzofuran derivatives showed in vitro anticancer potential [23]. Most of these compounds have been reported to have cytotoxic potential against many human cancer cell lines including leukemia (HL-60-TB and K-562), breast cancer (MDA-MB-435. MDA-N, BT-549 and MCF-7), human cervical cancer (HeLa) and human lung carcinoma (A549) cell lines [23, 28]. Based on the previous reports, we screened the anticancer potential of the three isolated dihydrobenzofuran derivative from *P. barbatum* against oral (CAL-27) and lung cancer (NCI-H460) cells. Lung and oral cancer was selected by considering their prevalence in Pakistan due to excessive smoking, tobacco and beetle nut chewing. Oral and lung carcinoma ranked 2nd and 3rd respectively out of top 10 cancer related malignancies in Pakistan [31]. Among isolated compounds **1** and **3** showed better anticancer activity against oral cancer cell line (CAL 27) with IC_50_ 48.52 ± 0.95 and 86.95 ± 4.39 respectively which was lower than the standard drug (5-Fluorouracil; Table 1). Whereas, these two compounds also showed anticancer activities against lungs cancer cells (NCI-H460) at higher doses as compared to control drug (Cisplatin; Table 1). Compound **1** was selected for further apoptosis analysis by observing morphological changes and FACS analysis considering its IC_50_ which was almost half the IC_50_ of standard drugs. After treatment with compound **1** at IC_50_ concentration cell morphology started to change after 24 h and with the passage of time cell detach from the surface with loss in cell shape suggesting apoptosis as compared to control. On the other hand, at higher concentration, i.e. IC_70_ cells were undergone apoptosis after 24 h (Fig. 3). Flow cytometry analysis (FACS) also correspond with the morphological data that after 48 h of treatment significant apoptosis (either early or late) and necrosis was observed (Fig. 4).

**Table 1:** Anticancer activity of compounds against oral cancer (CAL-27) and lung cancer (NCI H460) cell lines

Compound	IC_50_ ± SD (µM)	
	CAL-27	NCI H460
**5-Fluorouracil**	97.76 ± 3.44	–
**Cisplatin**	–	19 ± 1.24
**Compound 1**	48.52 ± 0.95	53.24 ± 1.49
**Compound 2**	> 250	198.05 ± 4.79
**Compound 3**	86.95 ± 4.39	93.34 ± 1.10

### Antiangiogenic studies

Chicken chorioallantoic membrane (CAM) is a low cost in vivo tumor biology model and is used widely for the tumor angiogenesis studies. Tumor growth depends on blood supply which in turn depends on the number of vessels. Hence, the inhibition of neovascularization is one of the targets. We recently reported dihydropyrimidine-based compounds that have potential to inhibit of new blood vessels formation [15]. Moreover, we have also employed CAM assay for the antitumor and anti-angiogenic potentials of crude extracts of some medicinal plants. In current research, we also studied the potential of our isolated dihydrobenzofuran derivatives on normal vascular development in chick embryos using CAM model. Normal saline was used as negative control. Dexamethasone and methotrexate was used as positive control [15].

**Fig. 5:**
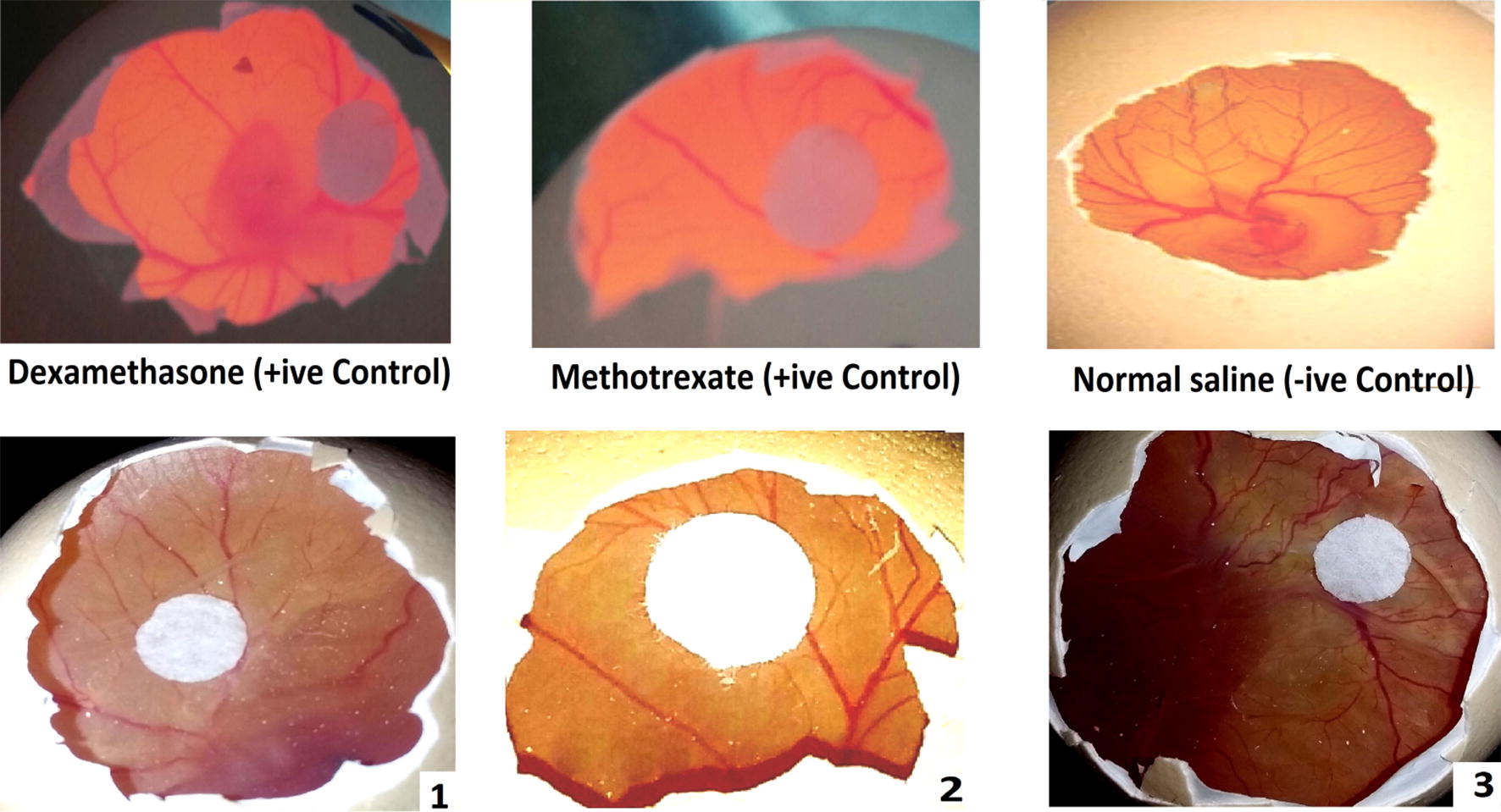
Anti-angiogenic activity of isolated dihydrobenzofuran derivatives along with positive and negative control. Positive control CAM was applied with dexamethasone and methotrexate

The antiangiogenic activities of the isolated compounds **1**, **2** and **3** were based on the number of blood vessels formed in the chorioallantoic membrane (CAM) of chick embryos. Results of anti-angiogenic assay of isolated compounds are shown in Table 2. Additional file 1: Table S3 showed the average number of blood vessels of each concentration in the CAM of chick embryo using different five concentration of each compound. Chick embryos treated with distilled water yielded the highest mean of 30.4 blood vessels formed. This indicates that water, being the negative control, is the least antiangiogenic among the five treatments. The compound **3** (IC_50_ = 57.7 ± 0.3 μM, Table 2), yielded moderate number of blood vessels with average mean of about 10.8. The compound **2** (IC_50_ = 13.4 ± 1.1 μM) yielded an average of 8.6 blood vessels formed. While, the compound **1** (IC_50_ = 8.2 ± 1.1 μM) yielded a mean of only 5.0 blood vessels which were most active against antiangiogenic activity as given in Fig. 5. The IC_50_ value was calculated via regression analysis of dose response curve by adding all the concentrations and percent activities (Figure S1 in Additional file 1).

**Table 2:** Results of anti-angiogenic assay of isolated compounds

Samples	IC_50_ (μM)
1	8.2 ± 1.1
2	13.4 ± 1.1
3	57.7 ± 0.3
Dexamethasone	33.0 ± 0.6
Methotrexate	0.90 ± 0.06

### Docking studies

In cancer research, cancer cell lines have emerged as an alternative tool for the study of biological mechanisms. However, to hypothesize a biomolecular target that help us to explore the mechanism is a difficult task. The standard drugs used in current research are cisplatin and 5-fluorouracil. Cisplatin is a chemotherapeutic agent that binds with DNA to form a bifunctional 1,2-intrastand cross link. While, 5-fluorouracil is a thymidylate synthase (TS) inhibitor that interrupt its action resulting in inhibition of TS and DNA replication. We extended our investigations and use docking studies to explore the mechanism of anticancer activity.

**Fig. 6:**
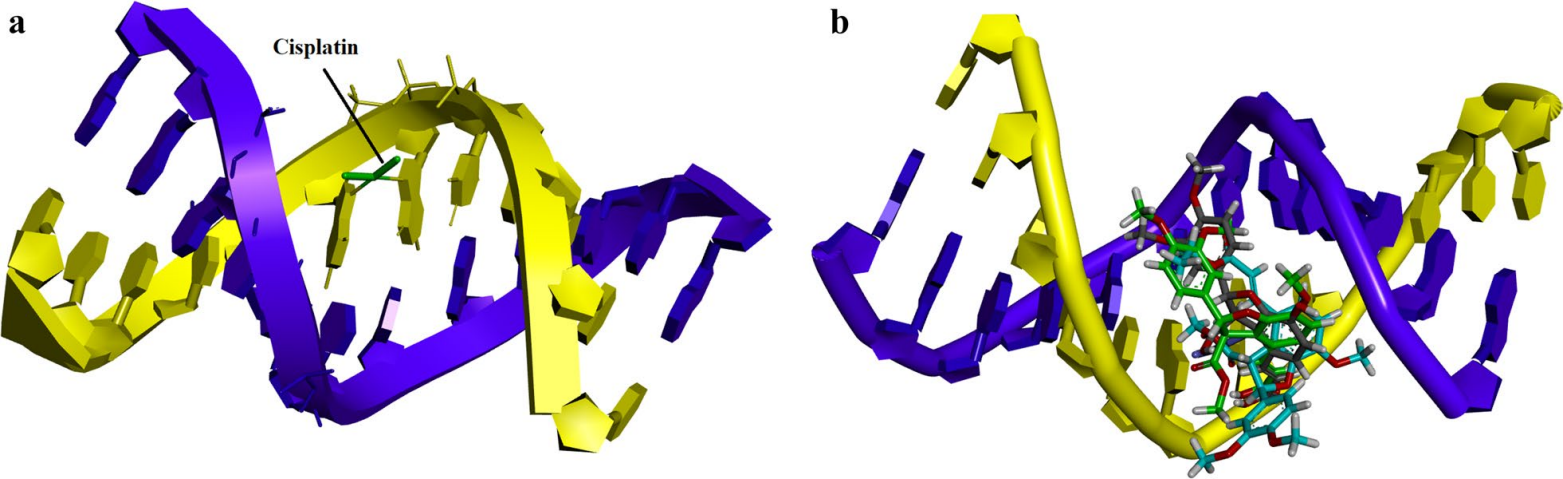
**a** Representation of the crystal structure of DNA duplex receptor was retrieved from Protein Data Bank (PDB code 3LPV) containg cisplatin 1,2-d(GpG) intra-strand cross-link. **b** Overlaid diagram of all the compounds targeting major groove in the cisplatin binding site

**Fig. 7:**
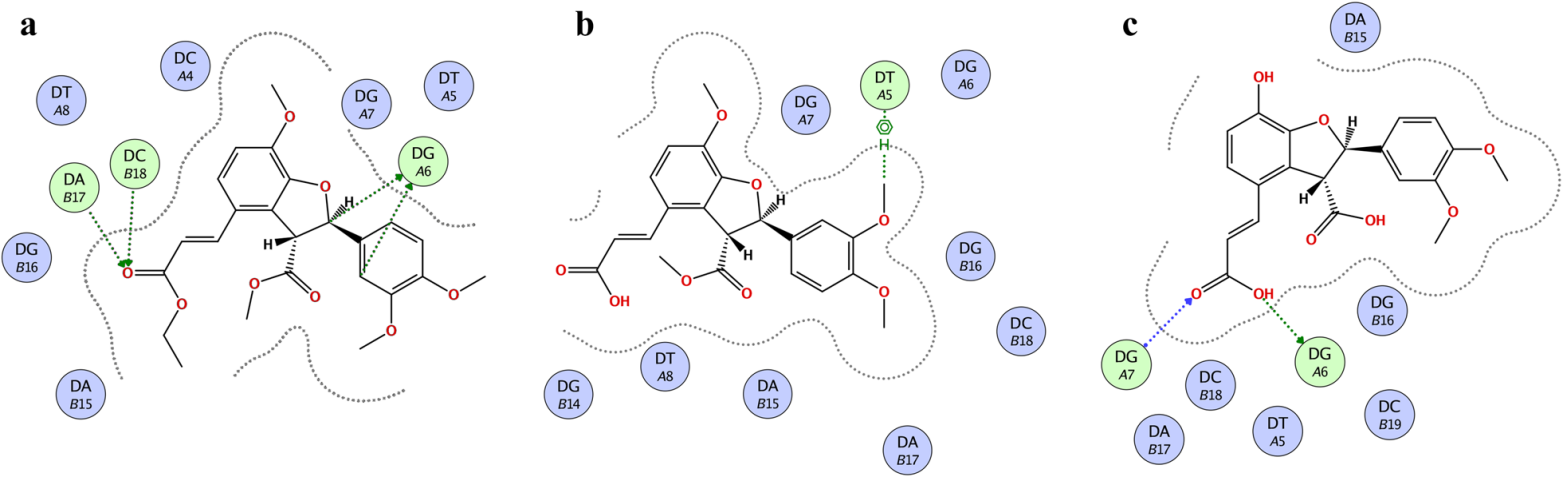
**a** Two-dimensional interaction plot of the synthesized compounds with DNA **a** compound **1**; **b** compound **2** and; **c** compound **3**

The crystal structure of DNA duplex receptor was retrieved from Protein Data Bank (PDB code 3LPV) (Fig. 6a). All these compounds targeted major groove in the cisplatin binding site (Fig. 6b). Carbonyl oxygen of ester of compound **1** forms hydrogen bond interactions (HBI) with cytosine and adenine (Fig. 7a). Methoxy group of compound **2** forms π–H interactions with thymine nucleoside (Fig. 7b). While hydroxyl group and carbonyl group of compound **3** forms HBI with guanine A6 and guanine A7 respectively (Fig. 7c). The computed binding affinity of compounds **1**–**3** are − 6.4355, − 5.9216, − 5.9376 kcal/mol respectively.

**Fig. 8:**
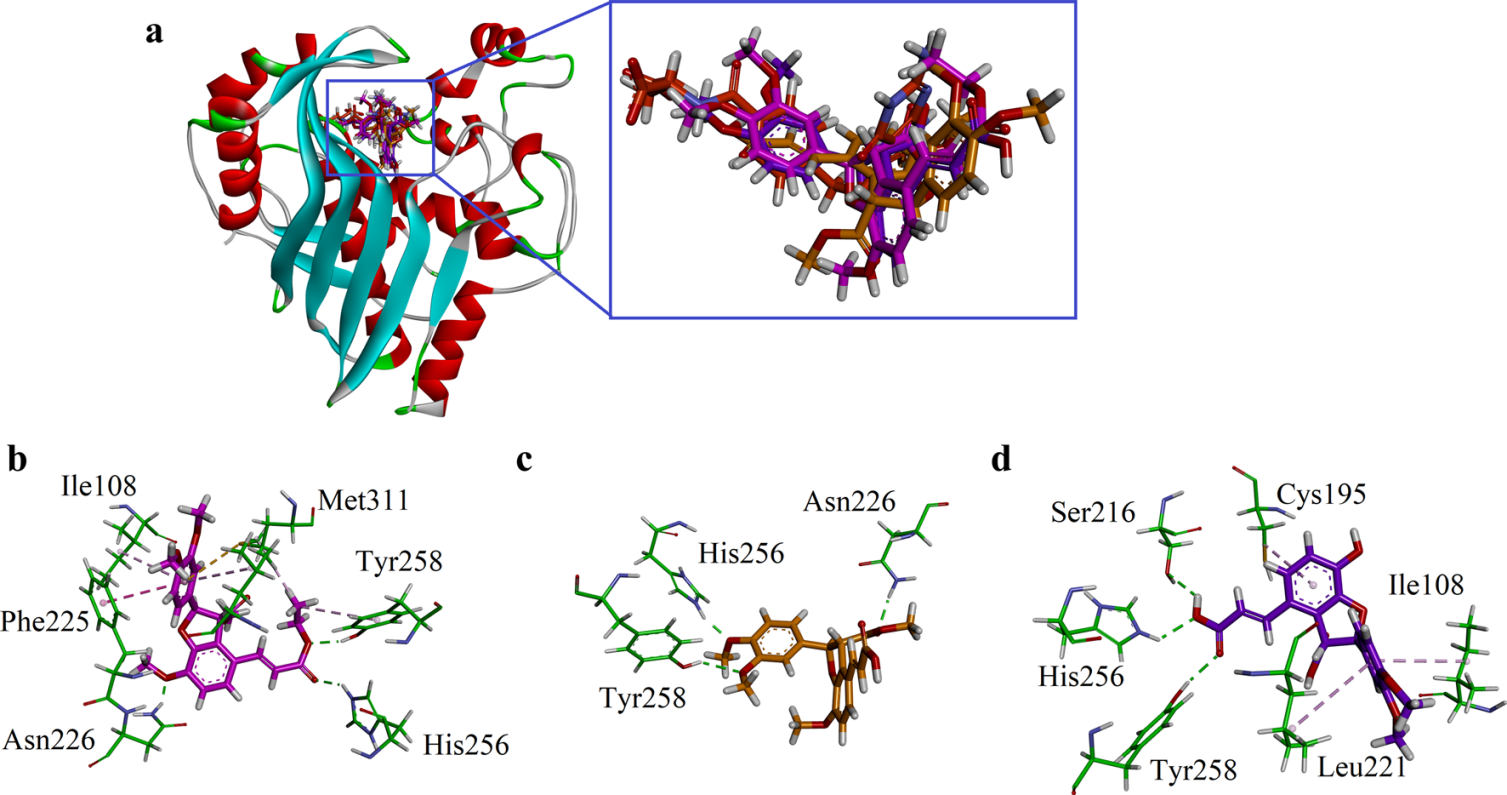
**a** The overlaid ribbon diagram of Native ligand (green), compound **1** (pink), compound **2** (yellow) and compound **3** purple) into the binding site of hTS (PDB code 1JU6); **b**–**d** Close-up depiction of the lowest-energy three-dimensional (3-D) docking pose of compounds **1**–**3** respectively

For the study of another target, thymidylate synthase (TS), its crystal structure retrieved from PDB (code 1JU6) with LYA as native ligand. The three-dimensional (3D) overlaid diagram of native ligand LYA and compounds **1**–**3** is shown in Fig. 8a. While, the 3D interaction diagram of compounds **1**–**3** is shown in Fig. 8b–d. Carbonyl oxygen of ester of compound **1** form HBI with His256. While oxygen atom of –OC_2_H_5_ forms HBI with Tyr258. Met311 for π–sulfur interactions with dimethoxyphenyl ring. Dimethoxyphenyl ring also forms π–π stacking interactions with Phe225. Another HBI was found between –OCH_3_ and Asn226. The ligand–enzyme complex also stabilize through π–alkyl interactions (Fig. 8b). For compound **2**, three HBI were found. The two OCH_3_ groups forms HBIs with His256 and Tyr258. While, carbonyl oxygen forms HBI with Asn226. Compound **3** also forms three HBIs and two π-alkyl interactions. Carboxylic acid group (HO–C=O) establishes HBIs with Ser216, His256 and Tyr258. Cys195, Ile108 and Leu221 forms π–alkyl interactions. The binding affinity of compounds **1**–**3** is − 7.9363, − 7.1656 and − 7.7879 kcal/mol respectively. Here, we can conclude that compound **1** has with two ester groups oriented into the binding site to show maximum interactions and lowest energy (most stable ligand enzyme complex).

## Conclusions

The phytochemical investigation of ethyl acetate fraction of *P. barbatum* resulted in the isolation of three new derivatives (1–3) of dihydrobenzofuran. Various spectroscopic techniques like mass spectrometry, UV, IR, 1D and 2D-NMR spectroscopy (^1^H-NMR, ^13^C-NMR, HMBC, NOESY) were employed for characterization of compounds (**1**–**3**). The structures of compound (**1**–**3**) were deduced to be methyl (2*S*,3*S*)-2-(3,4-dimethoxyphenyl)-4-((*E*)-3-ethoxy-3-oxoprop-1-en-1-yl)-7-methoxy-2,3-dihydrobenzofuran-3-carboxylate (1), (*E*)-3-((2*S*,3*S*)-2-(3,4-dimethoxyphenyl)-7-methoxy-3-(methoxycarbonyl)-2,3-dihydrobenzofuran-4-yl)acrylic acid (2) and (2*S*,3*S*)-4-((*E*)-2-carboxyvinyl)-2-(3,4-dimethoxyphenyl)-7-hydroxy-2,3-dihydrobenzofuran-3-carboxylicacid (3). Among the isolated compounds **1** and **3** were found to be more active against oral cancer cell lines than lung cancer cells as compared to standard drugs. Further investigations via in vivo antiangiogenic activity using Chicken chorioallantoic membrane (CAM) model showed that isolated compound **1** with IC_50_ of 8.2 ± 1.1 μM showed excellent antiangiogenic activity. Herein, we can conclude that in future, these compounds can be further studied to understand the mechanism of action involved in their anticancer potential.

## Additional file

**Additional file 1.** Supporting information.

## Abbreviations

UV: ultravoilet visible spectroscopy
IR: infrared spectroscopy
NMR: nuclear magnetic resonance spectroscopy
MTT: 3-(4,5-dimethyl-2-thiazolyl)-2,5-diphenyl-2-H-tetrazolium bromide
TLC: thin layer chromatography
MHz: mega hertz
HR-EI-MS: high resolution electron impact mass spectroscopy
s: singlet
d: doublet
t: triplet
q: quartet
dd: doublet of doublet
*J*: coupling constant
DMEM: Dulbecco’s Modified Eagle’s Medium
CAM: chorioallantoic membrane
HMBC: heteronuclear multiple-bond correlation
