# Supplementary material for: Isolation of dihydrobenzofuran derivatives from ethnomedicinal species Polygonum barbatum as anticancer compounds
Source: Biol Res. 2019 Jan 7;52:1. doi: 10.1186/s40659-018-0209-0 (PMC6322347; doi:10.1186/s40659-018-0209-0)
Supplement: Supplementary file 1 — Additional file 1. Supporting information. [file 40659_2018_209_MOESM1_ESM.docx]

***Additional Information***

**Isolation of** **dihydrobenzofuran derivatives from ethnomedicinal species *Polygonum barbatum* as anticancer compounds**

# Umar Farooq^1*^, Sadia Naz^1^, Afshan Shams^2^, Yasir Raza^3^, Ayaz Ahmed^2^, Umer Rashid^1**^, Abdul Sadiq^4,5***^

^1^Department of Chemistry, COMSATS University Islamabad, Abbottabad Campus, 22060 Abbottabad, Pakistan

^2^Dr. Panjwani Center for Molecular Medicine and Drug Research, International Center for Chemical and Biological Sciences, University of Karachi, Pakistan

^3^Department of Microbiology, University of Karachi, Pakistan

^4^Department of Pharmacy, University of Malakand, Chakdara, 18000 Dir (L), KP, Pakistan

^5^Department of Life Sciences & Chemistry, Faculty of Health, Jacobs University Bremen, 28759 Bremen, Germany

**Corresponding Authors:**

*Email [umarf@ciit.net.pk](mailto:umarf@ciit.net.pk);Contact: 0092(0)300 8956 781 (Umar Farooq),

**Email: [umerrashid@ciit.net.pk](mailto:umerrashid@ciit.net.pk);Contact: 0092(0)334 5171 999(Umer Rashid)

***Email:[sadiquom@yahoo.com](mailto:sadiquom@yahoo.com) Contact: +92(0)301 2297 102**(**Abdul Sadiq)

**Table S1**: ^1^H NMR (400 MHz, CD_3_OD), δ_H_ in ppm

| **Carbon No** | **Compound 1** | **Compound 2** | **Compound 3** |
| --- | --- | --- | --- |
|  | **^1^H-NMR**  **(δ_H_ ppm)** | **^1^H-NMR**  **(δ_H_ ppm)** | **^1^H-NMR**  **(δ_H_ ppm)** |
| 1 | - | - | - |
| 2 | - | - | - |
| 3 | - | - | - |
| 4 | - | - | - |
| 5 | 6.90 (1H, d, *J* = 9.1 Hz) | 6.88 (1H, d, *J* = 9.2 Hz) | 6.89 (1H, d, *J* = 8.8 Hz) |
| 6 | 7.30 (1H, d, *J* = 9.1 Hz) | 7.28 (1H, d, *J* = 9.2 Hz) | 7.27 (1H, d, *J* = 8.8 Hz) |
| 7 | 7.80 ((1H, d, *J* = 16.5 Hz) | 7.70 ((1H, d, *J* = 15.4 Hz) | 7.78 ((1H, d, *J* = 17.1 Hz) |
| 8 | 6.54 (1H, d, *J* = 16.5 Hz) | 6.50 (1H, d, *J* = 15.4 Hz) | 6.48 (1H, d, *J* = 17.1 Hz) |
| 9 | - | - | - |
| 10 | 4.30 (2H, q, *J* = 8.1 Hz) | - | - |
| 11 | 1.10 (3H, t, *J* = 8.1 Hz) | - | - |
| 1′ | - | - | - |
| 2′ | 6.80 (1H, d, *J* = 1.8 Hz) | 6.80 (1H, d, *J* = 1.9 Hz) | 6.74 (1H, d, *J* = 2.1 Hz) |
| 3′ | - | - | - |
| 4′ | - | - | - |
| 5′ | 6.61 (1H, d, *J* = 8.2 Hz) | 6.65 (1H, d, *J* = 8.8 Hz) | 6.64 (1H, d, *J* = 7.9 Hz) |
| 6′ | 6.63 (1H, dd, *J* = 8.2, 1.8 Hz) | 6.72 (1H, dd, *J* = 8.8, 1.9 Hz) | 6.68 (1H, dd, *J* = 7.9, 2.1 Hz) |
| 7′ | 5.92 (1H, d, *J* = 5.2 Hz) | 5.90 (1H, d, *J* = 4.8 Hz) | 5.91 (1H, d, *J* = 5.5 Hz) |
| 8′ | 4.68 (1H, d, *J* = 5.2 Hz) | 4.70 (1H, d, *J* = 4.8 Hz) | 4.74 (1H, d, *J* = 5.5 Hz) |
| 9′ | - | - | - |
| 4-OCH_3_ | 3.60 (3H, s) | 3.60 (3H, s) | - |
| 3′-OCH_3_ | 3.64 (3H, s) | 3.62 (3H, s) | 3.58 (3H, s) |
| 4′-OCH_3_ | 3.66 (3H, s) | 3.65 (3H, s) | 3.60 (3H, s) |
| 9′-OCH_3_ | 3.55 (3H, s) | 3.53 (3H, s) | - |

Table S2: ^13^C NMR (100 MHz, CD_3_OD), δ_C_ in ppm

| **Carbon No** | **Compound 1** | **Compound 2** | **Compound 3** |
| --- | --- | --- | --- |
|  | **^13^C-NMR**  **(δ_C_ ppm)** | **^13^C-NMR**  **(δ_C_ ppm)** | **^13^C-NMR**  **(δ_C_ ppm)** |
| 1 | 126.6 | 124.5 | 124.2 |
| 2 | 129.7 | 128.2 | 128.1 |
| 3 | 151.9 | 150.6 | 140.4 |
| 4 | 146.3 | 147.8 | 146.6 |
| 5 | 121.3 | 120.2 | 119.9 |
| 6 | 122.4 | 125.1 | 123.1 |
| 7 | 143.7 | 142.4 | 141.4 |
| 8 | 120.7 | 119.6 | 120.8 |
| 9 | 170.1 | 172.1 | 173.1 |
| 10 | 66.2 | - | - |
| 11 | 19.4 | - | - |
| 1′ | 135.9 | 134.7 | 135.7 |
| 2′ | 116.3 | 114.3 | 118.6 |
| 3′ | 153.2 | 152.7 | 153.1 |
| 4′ | 154.5 | 153.1 | 154.6 |
| 5′ | 118.6 | 117.6 | 116.7 |
| 6′ | 124.8 | 123.1 | 125.3 |
| 7′ | 82.4 | 84.1 | 84.4 |
| 8′ | 58.1 | 59.2 | 57.1 |
| 9′ | 174.6 | 174.1 | 175.6 |
| 4-OCH_3_ | 57.8 | 58.6 | - |
| 3′-OCH_3_ | 60.1 | 61.3 | 59.1 |
| 4′-OCH_3_ | 60.5 | 61.6 | 59.8 |
| 9′-OCH_3_ | 54.8 | 56.4 | - |

**Table S3:** Antiangiogenic activity of the test compounds based on blood vessels formed

| **Treatment** | **Number of blood vessels in CAM** | | | | | **Mean** |
| --- | --- | --- | --- | --- | --- | --- |
|  | **1** | **2** | **3** | **4** | **5** |  |
| **Normal saline (-ive control)** | 31 | 30 | 29 | 29 | 33 | 30.4 |
| **1** | 2 | 3 | 5 | 6 | 9 | 5 |
| **2** | 5 | 8 | 9 | 10 | 11 | 8.6 |
| **3** | 6 | 9 | 12 | 13 | 14 | 10.8 |
| **+ive control (Dexa)** | 3 | 5 | 6 | 10 | 12 | 7.2 |
| **+ive control methotrexate** | 0 | 2 | 3 | 4 | 5 | 2.8 |

**Figure S1:** IC_50_ value of compounds via methotrexate as well as dexamethasone used as standard
